# Stage IIIB ROS1-Positive NSCLC management: crizotinib + surgery + TCM achieves exceptionally prolonged PFS (a case report)

**DOI:** 10.3389/fphar.2026.1693269

**Published:** 2026-02-16

**Authors:** Yu Zhu, Hao Zhang, Zheng-xiang Han, Xu Zhu, Wan-ting Wang, Huai-yang Li, Shudong Zhu, Wei Shao

**Affiliations:** 1 Xuzhou Renci Hospital, Xuzhou, Jiangsu, China; 2 Affiliated Hospital of Xuzhou Medical University, Xuzhou, Jiangsu, China

**Keywords:** acupuncture, crizotinib, Huisheng oral liquid, NSCLC, PFS, ROS1 fusion, surgery, TCM

## Abstract

This article reports a case of a patient with small primary foci (11 mm*8 mm), highly malignant ROS1 fusion-positive non-small cell lung cancer (NSCLC) with mediastinal and supraclavicular lymph node metastases. After neoadjuvant crizotinib targeted therapy, the patient underwent lobectomy and lymph node dissection. Postoperatively, continuous targeted therapy combined with Traditional Chinese Medicine (TCM) adjuvant intervention achieved an extremely prolonged 62-month progression-free survival (PFS) without drug resistance or obvious side effects, compared to the median real-world PFS (rwPFS) of approximately 20 months for guideline-recommended crizotinib treatment. The last follow-up in August 2025 showed the patient remained in good survival status. This case highlights a novel strategy of neoadjuvant targeted downstaging followed by surgical resection for small-lesion, highly invasive NSCLC, as well as the potential value of integrating postoperative Chinese-Western medicine management for long-term survival and treatment tolerance.

## 1 Introduction

Non-small cell lung cancer (NSCLC) accounts for 85% of lung cancers (Nadal et al., 2024). ROS1 rearrangement is rare in NSCLC, with an occurrence of only 0.9%–2.6%, varying by study population and detection methods (Gendarme et al., 2022). Although first-line treatment with crizotinib provides clinical benefit in patients with ROS1-mutated lung cancer, resistant mutations and a high incidence of disease progression remain therapeutic challenges (Yun et al., 2020).

In general, patients with ROS1 fusion are sensitive to tyrosine kinase inhibitors (TKIs) such as crizotinib; however, cases with a small primary focus, high malignancy, and concomitant distant metastases have a poorer prognosis, with a median time to onset of brain metastases of 15.7 months with crizotinib treatment, in comparison to 12.5 months without crizotinib treatment (Solomon et al., 2016). The median real-world progression-free survival (rwPFS) ranges from 7.7 to 26.1 months, and the median overall survival (OS) ranges from 16.7 to 61.0 months (Nadal et al., 2024). The latest clinical practice guidelines recommend various treatment regimens for ROS1-rearranged advanced or metastatic NSCLC, including crizotinib, entrectinib, lorlatinib, etc. (National Comprehensive Cancer Network, 2025).

Notably, the guidelines have not addressed the application of targeted agents for downstaging followed by combined lesion excision and lymph node dissection. In this case, targeted therapy combined with surgery after downstaging provided clinical long-term benefit for a patient with stage IIIB NSCLC. Furthermore, systematic reports on the combined application of neoadjuvant targeted therapy, surgery, and adjunctive traditional Chinese medicine are lacking in the current literature. This case report underscores the feasibility of this integrated approach as a potential treatment strategy.

## 2 Case report

### 2.1 Clinical information

In February 2019, a 48-year-old female patient with no history of smoking and no family history of tumors underwent a routine physical examination. Chest computed tomography (CT) revealed a nodular shadow (IM19) measuring approximately 11 mm *8 mm in the posterior segment of the right upper lobe near the pleural surface. This result was not taken seriously by the patient.

**FIGURE 1:**
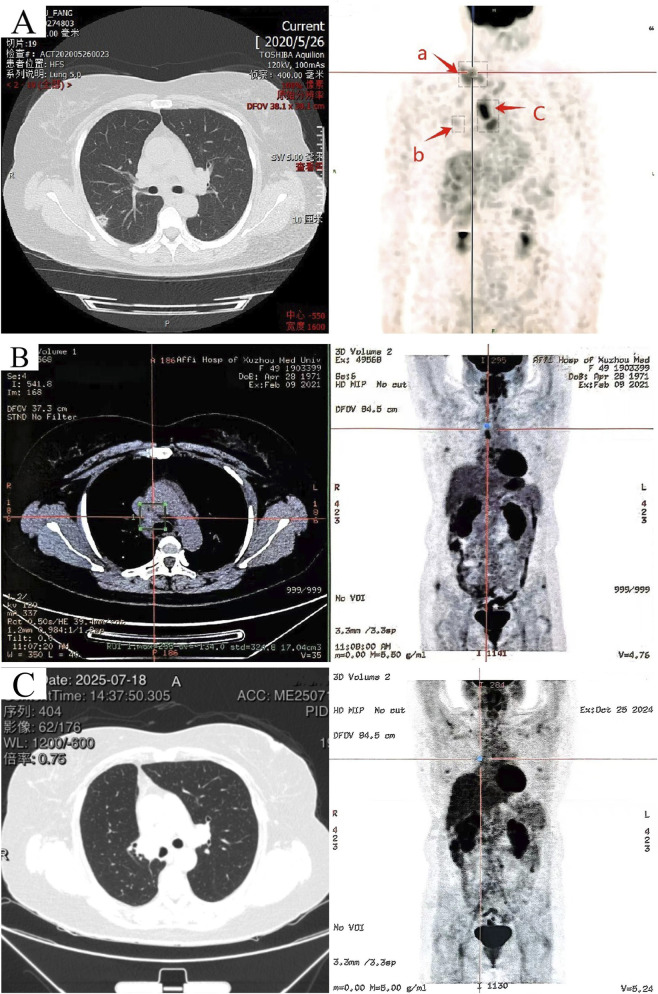
Imaging CT scans before crizotinib treatment, 8 months after crizotinib initiation, and 52 months postoperatively. **(A)** (June 2020) 64-row non-contrast chest CT and PET/SPECT-CT images: a nodular shadow in the right upper lobe (b), and focal abnormal elevation of glucose metabolism in multiple enlarged lymph nodes of the right supraclavicular fossa (a) and mediastinum (c). **(B)** (February 2021) PET-CT scan 8 months after crizotinib initiation: only hypermetabolic lymph nodes in the mediastinum. **(C)** Left: CT imaging from the follow-up in late July 2025. Right: PET-CT from October 2024 showing no increased glucose metabolism in mediastinal or clavicular lymph nodes.

**FIGURE 2:**
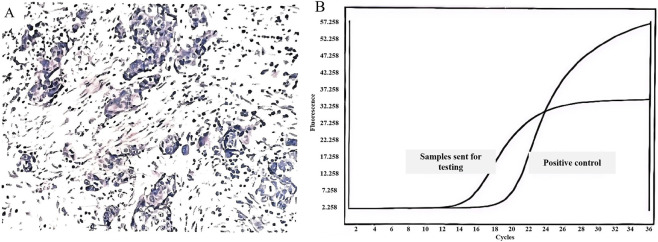
Characterization of Lung Cancer and ROS1 Genetic Alteration. **(A)** Pathology of right supraclavicular lymph node biopsy (June 2020): moderately differentiated adenocarcinoma; **(B)** Fluorescence quantitative qPCR profile: positive for ROS1 gene fusion.

In June 2020, the patient was admitted to the hospital due to a “right supraclavicular mass discovered for 5 days”. Cervical lymph node ultrasound findings were as follows: Multiple hypoechoic lesions were detected in levels 4 and 5 of the right cervical region, with the larger lesion measuring approximately 26*14 mm; multiple hypoechoic lesions were also identified in level 4 of the left cervical region, with the largest lesion being 16*10 mm.Immunohistochemistry results showed: CK7(+), CKpan (+), CK20(−), Vimentin (−), TTF-1 (+), NapsinA (−), P40 (−), P63 (−), GCDFP-15 (−), GATA3 (−), ER (2+), PR (−), HER2(−), E-cadherin (+), P120 (+), Ki67(+40%), adenocarcinoma, moderately differentiated, indictive of moderate-high proliferative activity. Perform whole-body FDG tumor scan, SPECT-PET/CT revealed the following: No focal areas of abnormally increased or decreased radiotracer uptake are identified within the brain. 1. A nodule in the right upper lobe with markedly increased fluorodeoxyglucose (FDG) uptake (Figure 1A, spot b), which is highly suspicious for malignancy; 2. Multiple enlarged lymph nodes in the right supraclavicular fossa (Figure 1A, spot a) and mediastinum (Figure 1A, spot c) with abnormal FDG avidity, consistent with lymph node metastasis. Comprehensive staging was T1bN3M0, stage IIIB (eighth AJCC edition). Molecular pathology identified ROS1 gene fusion (spliced exons 32/34), confirmed by qPCR analysis (Figure 2).

### 2.2 Course of treatment

The patient had multiple small nodules in both lungs and confirmed distant lymph node metastases, and was staged as IIIB. There was no indication for surgery, and the patient explicitly refused radiotherapy; therefore, crizotinib was administered clinically. For neoadjuvant therapy, crizotinib (250 mg twice daily) was given from 16 June 2020, to 14 February 2021 (a total of 8 months), with complete remission of lymph node metastases as assessed by RECIST 1.1 criteria.

Surgery: On 6 March 2021, a thoracoscopic right upper lobectomy with lymph node dissection was performed. Postoperative pathology of the right upper lung lobe revealed interstitial fibrous proliferation, small vessel proliferation, local collagenization, scattered chronic inflammatory cell infiltration, and carbon dust deposition accompanied by a multinucleated giant cell reaction. The lesion diameter was approximately 0.7 cm, and no definite tumor components were identified in all sampled tissues. No definite tumor metastasis was observed in lymph nodes from stations 2, 4, 7, 8, and 10 (0/1, 0/1, 0/2, 0/1 respectively).

Postoperative treatment: Following surgery, the patient has continued oral crizotinib (250 mg twice daily). From 8 October 2021, to 3 February 2024, the patient received a concurrent course of Huisheng Oral Liquid at 10 mL three times daily. The principal ingredients of this formulation include Leonuri Herba, Zanthoxyli Pericarpium, Carthami Flos, Hirudo, Sappan Lignum, Angelicae Sinensis Radix, among others (full composition of Huisheng Oral Liquid are provided in the Supplementary Material). Additionally, traditional Chinese acupuncture therapy has been utilized to alleviate medication-related side effects. The drug regimen, adverse events, and acupuncture management are summarized in Tables 1, 2. The traditional Chinese acupuncture therapy administered in this study was performed in accordance with the standardized protocols and point locations described in the authoritative textbook *Chinese Acupuncture Science (fifth edition)* (The People’s Health Press, 2019; ISBN: 978-7-117–27170-7).

**TABLE 1:** Patient treatment log: Drug regimen, adverse events & acupuncture management.

Treatment period	Medication regimen	Adverse event/Grade*	Acupoints intervened	Therapeutic effect
2020.06–2021.01	Crizotinib,250 mg bid po	Vomiting (Grade 1)	Hegu (LI4) \ Taichong (LR3) \ Taichong (LR3) \ Zhongwan (CV12) \ Liangmen (ST21) \ Neiguan (PC6)	Resolved immediately
		Vomiting (Grade 2)		Relieved within 3 days
2021.02–2021.05	Perioperative period			
2021.06–2021.09	Crizotinib,250 mg bid po	Nausea, vomiting, Diarrhea, Rash, chest tightness, Dyspnea, right lower limb edema (all grade 1–2)	No consultation, no intervention	-
2021.10–2024.1	Hui Sheng oral Liquid,10 mL bid po Crizotinib,250 mg bid po. (Five serial CEA* measurements were within the normal range:3–5 ng/mL)	Right lower limb edema (Grade 2)	Taixi (KI3) \ Fuliu (KI7) \ Siman (KI14) \ Sanyinjiao (SP6) \ Lougu (SP7) \ Yinlingquan (SP9)	Relieved within 3 days
		Chest tightness, Dyspnea (Grade 1–2)	Zhongfu (LU1) \ Tianfu (LU3) \ Taiyuan (LU9) \ Hegu (LI4) \ Zusanli (ST36) \ Taichong (LR3)	Resolved immediately
2024.02–2025.07	Discontinue the use of Huisheng oral Liquid, Crizotinib 250 mg bid po as monotherapy. (Five serial CEA measurements were elevated, ranging from 5.8–6.9 ng/mL (above the normal range).)	No significant adverse drug reactions were reported	Maintenance therapy: Acupuncture at KI3, SP6, ST36, LI4, LR3, CV12 using ping bu ping xie*. Frequency: Once/10 days	No discomfort was reported

*CEA (Normal range: <5 ng/mL).

*Adverse events graded per NCI CTCAE v5.0.

*ping bu ping xie: The needle was inserted at the point and stimulated using the even supplementation and drainage technique, characterized by a gentle, alternating twirling motion with uniform speed and amplitude.

**TABLE 2:** Acupuncture approaches for managing adverse effects of crizotinib.

CTCAE grade 1–2 adverse events	Acupoint	Acupuncture method	Needle manipulation techniques
leg edema	Taixi (KI3)	Puncture perpendicularly 0.3-0.5 cun*	Following pattern differentiation and treatment principles, after the arrival of deqi, needle manipulation can be applied. This includes the lift-thrust technique (tonifying by gentle thrusting and vigorous lifting; reducing by vigorous thrusting and gentle lifting) and/or the twirl-rotate technique (tonifying by rotating to the left; reducing by rotating to the right). The needle retention time is typically 15–20 min, which may be extended to 30 min for patients with deficiency patterns. Upon needle removal, withdraw the needles slowly and gently to avoid bleeding or discomfort
	Fuliu (KI7)	Puncture perpendicularly 0.8-1.0 cun	
	Siman (KI14)	Puncture perpendicularly 0.8-1.2 cun	
	Sanyinjiao (SP6)	Puncture perpendicularly 0.5-1.0 cun	
	Lougu (SP7)	Puncture perpendicularly 1.0-1.5 cun	
	Yinlingquan (SP9)	Puncture perpendicularly 1.0-1.5 cun	
chest tightness and dyspnea	Zhongfu (LU1)	Oblique puncture outward 0.5-0.8 cun	
	Tianfu (LU3)	Puncture perpendicularly 0.5-1.0 cun	
	Taiyuan (LU9)	Puncture perpendicularly 0.2-0.3 cun	
	Hegu (LI4)	Puncture perpendicularly 0.5-1.0 cun	
	Zusanli (ST36)	Obliquely puncture 0.5-0.8 cun	
	Taichong (LR3)	Puncture perpendicularly 0.5-1.0 cun	
Vomiting	Hegu (LI4)	Puncture perpendicularly 0.5-1.0 cun	
	Taichong (LR3)	Puncture perpendicularly 0.5-1.0 cun	
	Zusanli (ST36)	Puncture perpendicularly 0.5-1.0 cun	
	Zhongwan (CV12)	Puncture perpendicularly 0.5-1.0 cun	
	Liangmen (ST21)	Puncture perpendicularly 0.5-0.8 cun	
	Neiguan (PC6)	Puncture perpendicularly 0.5-1.0 cun	

*When the patient flexes the middle joint of the middle finger, the distance from the fingertip to the proximal interphalangeal crease is defined as one cun, approximately equivalent to the width of the patient’s thumb interphalangeal joint, usually ranging from 1.5 to 3 cm (varying with individual body size).

This table documents the treatment administered to the patient in this specific case and is for reference only. The selection of acupuncture points and needle manipulation techniques must be determined based on an individualized assessment of the patient’s condition.

### 2.3 Follow-up visit

As of August 2025, the progression-free survival (PFS) has reached 62 months, with no signs of recurrence or metastasis. The treatment is well-tolerated, and there is no indication of poor overall survival. According to the Quality of Life Questionnaire Core 30 (QLQ-C30), the total health status score is 83.33, the physical functioning score is 86.67, and the role functioning score is 83.33 (Cocks et al., 2023; Zang et al., 2025).

## 3 Discussion

Lung cancer and its targeted therapies are among our research interests (Xiao et al., 2024; Ai et al., 2018). Our studies have demonstrated that drug resistance following targeted therapy remains a common key challenge in the treatment of advanced tumors (Li et al., 2025b; Zhu et al., 2013). In this study, we addressed this critical issue in the management of advanced or metastatic NSCLC with ROS1 rearrangement through an innovative approach, achieving notable success.

In this case, a patient with initially unresectable stage IIIB ROS1-fusion lung adenocarcinoma (with distant lymph node metastasis) achieved a major pathological response after 8 months of neoadjuvant monotherapy with crizotinib, which inhibits the ROS1 fusion signaling pathway. This resulted in significant reduction of metastatic lymph node size and highly probable pathological complete response. This demonstrates that neoadjuvant targeted therapy substantially reduced tumor burden, thereby creating conditions for curative surgery. Postoperative pathology further confirmed the depth of therapeutic response. This outcome robustly validates crizotinib’s high efficacy against ROS1-positive tumors, consistent with prior pivotal clinical trial data (Shaw et al., 2014). This case offers a real-world demonstration of the “targeted neoadjuvant therapy followed by surgery' treatment pathway for a patient with locally advanced, ROS1 rearrangement-positive disease.

Notably, the 62-month PFS observed in this case far exceeds the previously reported median rwPFS range of 7.7–26.1 months (Nadal et al., 2024), suggesting that our neoadjuvant targeted therapy may significantly improve the prognosis of patients with locally advanced disease.

Interestingly, in a patient with resectable stage IIIA NSCLC harboring an LDLR-ROS1 fusion, adjuvant treatment with crizotinib also yielded a favorable survival benefit (Chen et al., 2022). In that case, postoperative targeted therapy showed good efficacy: clinical and radiological follow-ups revealed no evidence of progression or recurrence, with recurrence-free survival exceeding 29 months. Our case demonstrated the use of crizotinib in treating metastatic NSCLC, where remission of distant lymph node lesions was followed by radical surgery; the patient achieved a 62-month progression-free survival. Together with the aforementioned case, these findings form a comprehensive strategy applicable to both resectable and metastatic NSCLC. Even more interestingly, the NSCLC subtype reported in our case, despite exhibiting a more aggressive nature, is associated with more prolonged survival.

In the management of non-small cell lung cancer (NSCLC) in this case, acupuncture was used as an adjuvant supportive intervention, with its role primarily focused on alleviating treatment-related side effects (Garcia et al., 2013). Specifically, TCM acupuncture was applied to relieve drug-induced adverse effects such as nausea, vomiting, and edema caused by crizotinib administration, thereby preventing potential hepatic and renal impairment that might result from the co-administration of other oral medications. Acupuncture was not intended to directly kill tumor cells. However, whether it directly contributed to cancer cell death in this case remains unclear, though plausible.

It should be noted that there is currently no high-quality research demonstrating that acupuncture can directly inhibit the proliferation or metastasis of NSCLC cells. For example, in an exploratory study on acupuncture combined with immune checkpoint inhibitors in the treatment of advanced non-small cell lung cancer, reports showed that there was no statistically significant difference in the objective response rate (ORR) between the acupuncture group and the control group (P > 0.05) (Zhou et al., 2023).

However, theoretically, acupuncture can regulate inflammatory factors and activate immune cells. For instance, with regard to acupuncture at Zusanli (ST36) as described in this report, relevant studies have demonstrated that such acupuncture therapy can reduce pro-inflammatory factors (IL-6, PGE2), increase anti-inflammatory factors (e.g., IL-10), alleviate inflammation in the tumor microenvironment, and elevate the CD4^+^/CD8^+^ ratio. These effects can improve patients' effective survival rate through immune regulation (Ainiwa et al., 2025). Therefore, the acupuncture treatment in this study may produce such effects and may be associated with the current 62-month progression-free survival achieved following crizotinib and surgical treatment. However, the immune-modulating mechanism is a hypothesis based on literature, as no specific immune panels were performed for this case.

Additionally, the patient received Huisheng Oral Liquid in combination with crizotinib therapy following surgery. During the treatment period from October 2021 to December 2023, the patient’s tumor markers CEA levels remained largely within normal reference ranges. However, following discontinuation of Huisheng Oral Liquid, the patient’s CEA levels exhibited a persistent upward trend. Combined with concurrent imaging monitoring results, this temporal pattern suggests Huisheng Oral Liquid may be associated with maintaining stable tumor markers or prolonging survival in this patient.

This observation aligns with conclusions from some previous studies. For instance, a retrospective cohort study indicated that continuous use of Huisheng Oral Liquid for ≥3 months in patients with stage II-III non-small cell lung cancer may be associated with higher 2-year survival rates, with the survival benefit increasing over time (Wang et al., 2021).

TCM likely played a supportive role as well as likely to act as the primary anti-tumor agent. There is no evidence to rule out either possibility yet. It must be emphasized that the observed clinical associations are descriptive phenomena and do not establish causality; elevated CEA levels and prolonged survival may be influenced by multiple factors, including natural tumor progression, unobserved variables, or individual differences. Thus, the specific role and potential antitumor effects of Huisheng Oral Liquid require validation in prospective, rigorously designed clinical trials.

Another point that deserves special mention is that Crizotinib metabolism *in vivo* is heavily dependent on the cytochrome P450 enzyme system, with CYP3A4 serving as the predominant metabolizing enzyme. This metabolic profile renders its plasma concentration highly susceptible to interactions with concomitant medications or natural products (Wang et al., 2024). Huisheng Oral Liquid, a compound herbal formula composed of 34 medicinal ingredients, possesses a complex chemical profile. Given the complexity of its composition, its potential enzyme modulatory effects warrant further investigation. Taking one of its constituent herbs, Angelica sinensis, as an example, a pharmacological study in a rat model found that consecutive administration of its aqueous extract for 7 days significantly induced hepatic CYP2E1 and CYP3A enzyme activities (Tang et al., 2006). However, another study yielded contradictory findings, in a liver microsome incubation system, Angelica sinensis exhibited inhibitory effects on CYP3A4 (Li et al., 2020). The current research findings regarding the interaction between Angelica sinensis and CYP3A4 are inconsistent. Therefore, *in vitro* results from single-herb components cannot be directly extrapolated to the *in vivo* effects of complex herbal formulations. Within Huisheng Oral Liquid, the various constituents may exhibit synergistic, antagonistic, or counteracting interactions, and the overall modulatory effect on CYP3A4 may differ significantly from that of any single herb. In this case, no severe CYP3A4-mediated drug-drug interactions were observed with the combination of crizotinib and Huisheng Oral Liquid. However, this finding does not entirely rule out potential risks, as considerable inter-individual variability may significantly influence drug metabolism processes (Zhou et al., 2009).

Last but not least, in the 2025 guidelines, crizotinib is listed as one of the first-line targeted agents for ROS1-rearranged NSCLC, and together with entrectinib and repotrectinib, forms the preferred treatment options. Compared with the latter two agents, crizotinib may have advantages in terms of PFS and ORR but exhibits weaker central nervous system (CNS) activity. This was why we chose crizotinib over the other two, and the results have confirmed that the original goals were achieved. However, in patients with brain metastases, the latter two targeted drugs should be prioritized, as they may offer greater advantages in CNS efficacy and post-resistance treatment.

As a single-case report, this study has the following inherent limitations: First, the single-subject design lacks controls, making it impossible to extrapolate results or establish causality. Second, since the treatment involved a comprehensive strategy, we cannot distinguish the specific contributions of targeted therapy, surgery, and traditional Chinese medicine. Third, the observed efficacy cannot exclude the influence of the tumor’s intrinsic biological characteristics, the patient’s high sensitivity to TKIs, or other unknown confounding factors. Moreover, a broader panel of diagnostic tests may be conducted to gain deeper insights into the intrinsic nature of the cancer, including the detection of PD-L1 expression levels. (Notably, PD-L1-targeted therapy is not recommended as a first-line intervention in the context of our study cohort.). Consequently, the conclusions drawn from this case represent preliminary indications only and require validation through further research.

## 4 Conclusion

Our case report presents a promising treatment option for patients with ROS1-fusion stage IIIB NSCLC and offers a novel reference for effective management of unresectable locally advanced NSCLC. For patients with ROS1 fusion-positive NSCLC, the integrative approach of neoadjuvant targeted therapy, surgical resection, and postoperative Chinese-Western medicine was associated with longer survival and quality-of-life benefits. Moving forward, we aim to expand the sample size to further validate this treatment model and explore the molecular mechanisms underlying the potential efficacy of this traditional Chinese medicine intervention.

## Data Availability

The original contributions of this study are included in the article. Further inquiries can be directed to the corresponding authors.
