# Supplementary material for: Stage IIIB ROS1-Positive NSCLC management: crizotinib + surgery + TCM achieves exceptionally prolonged PFS (a case report)
Source: Front Pharmacol. 2026 Feb 16;17:1693269. doi: 10.3389/fphar.2026.1693269 (PMC12950267; doi:10.3389/fphar.2026.1693269)
Supplement: Supplementary file 1 [file Supplementaryfile1.docx]

**1 Composition and Complete Weight Formulation of the 34 Herbal Medicines in Huisheng Oral Liquid (per 1000 ml of finished product)**

| **Order** | **Common Name** | **Scientific Name** | **Processing methods** | **Weight (g)** |
| --- | --- | --- | --- | --- |
| 1 | Motherwort | *Leonurus japonicus* Houtt. | - | 140 |
| 2 | Safflower | *Carthamus tinctorius* L. | - | 17.5 |
| 3 | Sichuan Pepper | *Zanthoxylum bungeanum* Maxim. | Carbonized* | 17.5 |
| 4 | Leech | *Whitmania pigra* Whitman | Processing* | 17.5 |
| 5 | Chinese Angelica | *Angelica sinensis* (Oliv.) Diels | - | 35 |
| 6 | Sappan Wood | *Caesalpinia sappan* L. | - | 17.5 |
| 7 | Burreed Tuber | *Sparganium stoloniferum* Buch.-Ham. | Aquafried vinegar* | 17.5 |
| 8 | Radde Anemone Rhizome | *Anemone raddeana* Regel | - | 17.5 |
| 9 | Chuanxiong Rhizome | *Ligusticum chuanxiong* Hort. | - | 17.5 |
| 10 | Rosewood | *Dalbergia odorifera* T. Chen | - | 17.5 |
| 11 | Nutgrass Rhizome | *Cyperus rotundus* L. | Aquafried vinegar | 17.5 |
| 12 | Ginseng | *Panax ginseng* C.A.Mey. | - | 52.5 |
| 13 | Galangal | *Alpinia officinarum* Hance | - | 17.5 |
| 14 | Turmeric | *Curcuma longa* L. | - | 10.5 |
| 15 | Myrrh | *Commiphora myrrha* Engl. | Aquafried vinegar | 17.5 |
| 16 | Bitter Apricot Kernel | *Prunus armeniaca* L. var. *ansu* Maxim. | Stirfried* | 26.25 |
| 17 | Rhubarb | *Rheum palmatum* L. | - | 70 |
| 18 | Perilla Fruit | *Perilla frutescens*(L.)Britt. | - | 17.5 |
| 19 | Fennel | *Foeniculum vulgare* Mill. | Stir-frying with salt* | 26.25 |
| 20 | Peach Kernel | *Prunus persica*(L.)Batsch | - | 26.25 |
| 21 | Flying Squirrel Feces | *Trogopterus xanthippes* Milne-Edwards | Aquafried vinegar | 17.5 |
| 22 | Gadfly | *Tabanus mandarinus* Schiner | - | 17.5 |
| 23 | Turtle Shell | *Pelodiscus sinensis*(Wiegmann) | - | 140 |
| 24 | Clove | *Syzygium aromaticum*(L.) Merr. & L.M.Perry | - | 21.25 |
| 25 | Corydalis Rhizome | *Corydalis yanhusuo* W. T. Wang | Aquafried vinegar | 17.5 |
| 26 | White Peony Root | *Paeonia lactiflora* Pall. | - | 35 |
| 27 | Cattail Pollen | *Typha angustifolia* L. | Carbonized | 17.5 |
| 28 | Frankincense | *Boswellia sacra*Flueck. | Aquafried vinegar | 17.5 |
| 29 | Dried Lacquer | *Toxicodendron vernicifluum*(Stokes)F. A. Barkl. | Calcined* | 17.5 |
| 30 | Evodia Fruit | *Tetradium ruticarpum*(A.Juss.) T.G.Hartley | Boiled licorice* | 17.5 |
| 31 | Asafoetida | *Ferula sinkiangensis* K.M.Shen | - | 17.5 |
| 32 | Cassia Bark | *Cinnamomum cassia* Presl | - | 17.5 |
| 33 | Mugwort Leaf | *Artemisia argyi* H.Lév.&Vaniot | Aquafried* | 17.5 |
| 34 | Prepared Rehmannia Root | *Rehmannia glutinosa* Libosch. | - | 35 |

**Adjuvants: Polysorbate 80 (2 g), Sodium Cyclamate (2 g), to prepare 1000 ml.**

*Carbonized: Take the materials to be processed, place them in a heated pan, and stir-fry with strong fire until the surface turns charred-black and the interior becomes charred-brown, or to the specified degree. Spray a small amount of clean water, extinguish any sparks, remove, and air-dry.

Processing: Take the cleaned materials to be processed, and according to the stir-frying method, fry them with talc until slightly expanded. Then cut into sections and dry.

Aquafried vinegar :Take the materials to be processed, mix thoroughly with vinegar, allow to suffuse thoroughly, place in a stir-frying container, and stir-fry until the specified degree is achieved. Remove and allow to cool. For vinegar processing, rice vinegar is used. Unless otherwise specified, use 20 kg of rice vinegar per 100 kg of materials to be processed.

Stirfried :Take the materials to be processed, place them in a stir-frying container, and heat with mild fire until the specified degree is achieved. Remove and allow to cool. For those requiring stir-frying to scorch, generally use moderate fire to stir-fry until the surface is charred-brown and the cross-section is scorched-yellow. Remove and allow to cool. For materials prone to ignition during scorching, a small amount of clean water may be sprayed before stir-frying to dryness.

Stir-frying with salt :Mix the materials to be processed thoroughly with brine, allow to suffuse thoroughly, place in a stir-frying container, and heat with mild fire. Stir-fry until the specified degree is achieved, then remove and allow to cool. For salt processing, edible salt is used. It should first be dissolved in an appropriate amount of water, filtered, and set aside for use. Unless otherwise specified, use 2 kg of salt per 100 kg of materials to be processed.

Calcined :Break the materials to be processed into small pieces, place them in a suitable container, and calcine until they become fragile or bright red throughout. Remove, allow to cool, and grind into powder.

Boiled licorice :Sort the materials to be processed by size. According to the specifications under each individual processing monograph, boil thoroughly with licorice until no white core is visible in the center when cut open. Remove, air-dry until 60% dry, slice, and dry.

Aquafried :Aquafrying refers to the method where materials to be processed are mixed and moistened with liquid adjuvants, then stir-fried to a specified degree.
